# Study on Degradation Law and the Equivalent Thickness Model of Steel Subjected to Sulfate Corrosion

**DOI:** 10.3390/ma16124320

**Published:** 2023-06-11

**Authors:** Tong Zhang, Qian Xu, Fan Yang, Shan Gao

**Affiliations:** 1 School of Resources and Civil Engineering, Liaoning Institute of Science and Technical, Benxi 117004, China; zt_1987_zt@163.com (T.Z.);; 2 School of Civil Engineering, Liaoning Technical University, Fuxin 123000, China; 3 Key Lab of Structures Dynamic Behavior and Control of the Ministry of Education, Harbin Institute of Technology, Harbin 150000, China

**Keywords:** mechanical properties, degradation law, equivalent thickness, sulfate corrosion

## Abstract

In order to study the variation of mechanical properties of steel under acid rain corrosion conditions in northern China, monotonic tensile tests were conducted on Q235 steel with a thickness of 3.0 mm and 4.5 mm using a method of artificially prepared simulated acid rain solution for indoor accelerated corrosion. The results show that the failure mode of corroded steel standard tensile coupon includes normal fault and oblique fault. The failure patterns of the test specimen show that the thickness of the steel and corrosion rate affected the corrosion resistance. Larger thicknesses and lower corrosion rates will delay the failure mode of corrosion on steel. The strength reduction factor (*R*_u_), deformability reduction factor (*R*_d_) and energy absorption reduction factor (*R*_e_) decrease linearly with the increasing corrosion rate from 0% to 30%. The results are interpreted also from the microstructural point of view. The number, size, and distribution of the pits are random when the steel is subjected to sulfate corrosion. The higher the corrosion rate, the clearer, denser, and more hemispherical the corrosion pits. The microstructure of steel tensile fracture can be divided into intergranular fracture and cleavage fracture. As the corrosion rate increases, the dimples at the tensile fracture gradually disappear and the cleavage surface gradually increases. An equivalent thickness reduction model is proposed based on Faraday’s law and the meso-damage theory.

## 1. Introduction

Steel structures and steel-concrete composite structures have been widely used in engineering structures due to their high strength and ductile behavior [1,2,3,4]. However, steel easily suffers from environmental corrosion during its service life [5], which is known as ‘corrosion destruction’ all over the world [6]. The mechanical properties of steel are the key parameters to evaluate the strength of these structures and assess the remaining service life of corroded steel through its thickness in design codes [7,8,9,10]. With the increasing number of steel structures in service, it is necessary to study the degradation law of mechanical properties of steel tubes subjected to corrosion.

Corrosion media and wetting time are the two most important environmental factors affecting steel corrosion. Marine atmospheres (mainly Cl^−^) and industrial atmospheres (mainly SO_2_) have the most severe corrosion on steel, the widest distribution, and the largest losses. In recent years, relevant research work has been carried out on the degradation law of mechanical properties of corroded steel. In order to analyze the effect of chloride ions on the atmospheric corrosion rate of carbon steel, Ma [11] exposed Q235 steel to a marine atmospheric environment, and evaluated the effect of chloride ions on the protective characteristics of the rust layer through an infrared spectrum, SEM-EDAX analysis, linear polarization resistance and electrochemical impedance spectroscopy (EIS). The results showed that chloride ions affected the corrosion rate and the morphology and composition of the rust layer. Krzysztof [12] used the random field method to establish the degradation model of a corroded steel plate and used explicit dynamics to conduct nonlinear finite element analysis. The analysis showed that the irregularity of the steel plate surface after corrosion was one of the main reasons for the decline of the mechanical properties of the steel plate. Garbatov [13] carried out tensile tests on the steel plate specimens which were cut from the corroded box girder in the real seawater environment, and then obtained the mechanical properties of the specimens, and established the regression equation of the mechanical properties degradation of the corroded steel plate. Kong [14] carried out the neutral salt spray accelerated corrosion test on 10 groups of Q235 steel specimens, measured the three-dimensional data of the surface morphology and corrosion depth of the corroded steel plate with the three-dimensional non-contact surface topography instrument, studied the probability characteristics of the corrosion depth, analyzed the relationship between the average deviation and standard deviation of the corrosion depth and the corrosion rate, and proposed the autocorrelation function model of the corrosion depth random field model. Melchers [15] presented a probabilistic model for “at-sea” immersion corrosion of mild and low alloy steels based on fundamental physiochemical corrosion mechanics. Jie [16] formed a conical blind hole by mechanical drilling and milling to simulate corrosion pits. They studied the influence of the shape, depth and distribution of corrosion pits on the mechanical properties of steel. Finally, a multiple regression analysis was conducted to obtain a prediction model for elongation. Xu [17] established a modified constitutive model of corroded Q235 steel through a salt spray accelerated corrosion test and obtained the relationship between model control parameters and corrosion rate.

In addition to corroded steel, the mechanical properties of corroded reinforcement were also studied [18,19,20,21,22]. It was found that with increasing duration of exposure to a corrosive environment, the steel mass loss increases appreciably. In addition, a significant reduction of the tensile mechanical properties and ductility of the material was observed. It was noted that the degree of corrosion strongly affected the mechanical properties of the steel, particularly the ultimate stress and strain. Li [23] derived a generalized equivalent stress-strain equation for the corroded steel bars, which considers the pitting corrosion using a bilinear elastoplastic constitutive equation. The bond behavior of corroded reinforcing steel bars in concrete was also studied [24,25,26].

In industrial atmospheres, SO_2_ is adsorbed onto the surface of steel and combined with a liquid film to form H_2_SO_3_, which is then oxidized to H_2_SO_4_. It reacts with Fe to form FeSO_4_, which in turn hydrolyzes to produce trace amounts of H_2_SO_4_, continuing to corrode the steel substrate, forming an acid cycle locally. Yang [27] conducted the corrosion fatigue experiment on the CT specimen of Q420B under artificial acid rain spray. Based on the Paris model, a P-da/dN-ΔK model of corrosion fatigue crack growth rate considering the randomness of the model parameters and the volatility of da/dN data is also derived. Zuo [28] investigated the effects of pH value, chloride ion concentration and alternation of wetting and drying time in acid rain on the corrosion of 35CrMn and Q235 steel. The results showed that the corrosion rate of 35CrMn and Q235 steel increased with decreasing pH values of the simulated acid rain, whereas the corrosion potential of 35CrMn and Q235 steel became more negative.

In existing research, the main focus is on the effect of corrosion on the mechanical properties of steel, but the functional relationship between the equivalent reduction thickness of steel and corrosion time has not yet been accurately given. In addition, the composition of the atmospheric environment has a great impact on the corrosion of steel. Within the same corrosion time, the mechanical properties of corroded steel in different areas also differ. In view of the dispersion of mechanical properties of corroded steel, the evaluation of the mechanical properties of corroded steel in specific areas can only be based on local corrosion data to establish a more accurate corrosion model.

The study intends to establish an equivalent thickness model for the Q235 steel subjected to sulfate corrosion. In the work, the results of an experimental survey on artificially corroded steel are reported. The effect of corrosion on the mechanical properties of steel tensile coupons is tested and discussed. The results are interpreted also from the microstructural point of view. Simultaneously, the deterioration equations for the mechanical properties of the corroded steel are defined, which include the ultimate tensile strength reduction factor, deformability reduction factor, and energy absorption reduction factor. The equivalent reduced thickness equation considering sulfate corrosion rate is also proposed, which can be used by researchers in further studies and engineers in practice.

## 2. Materials and Methodology

### 2.1. Solution Preparation

The corrosion process was accelerated by adopting DC under ambient temperature. According to statistical pH values of rainwater from the year 2007 to 2018 [29], the pH values were adjusted to 4.50 by HNO_3_, which belonged to the range of average value. The artificial corrosion solution was composed of three chemical compounds: (1) Ca(NO_3_)_2_ (0.143 g·L^−1^); (2) Na_2_SO_4_ (0.251 g·L^−1^); and (3) NH_4_Cl (0.038 g·L^−1^). This corrosion solution contained SO_4_^2−^, Cl^−^, NO_3_^−^, Ca^2+^, NH_4_^+^, and Na^+^ to simulate the precipitation components of the district in refs [30,31]. In order to keep the stability of the corrosion solution of pH value, it was measured per 12 h. If the pH value is beyond the scope of 4.2–4.8, the corrosion solution needed to be replaced. The reaction rate is determined by the diffusion of H^+^ to the interface, thus the pH value is checked twice a day.

**Figure 1:**
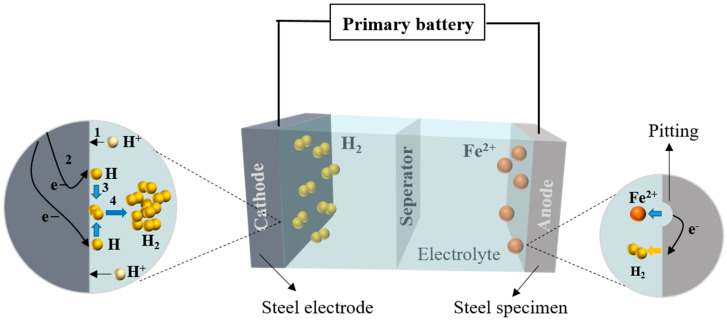
Principle of steel corrosion in an acid electrolyte.

Figure 1 shows that the H_2_ (g) is generated from the cathode, which causes overpotential. Since the migration speed of hydrogen ions is faster than that of other ions, acid medium HNO_3_ is adopted to supply the ions in the electrode. Using an acid medium can avoid the overpotential of other ions and reduce the internal resistance of the electrolyte, which can result in the slow decaying and stability of current electron density.

The tested tensile specimen in acid electrolyte presented pitting corrosion. It can be seen in Figure 1 that the oxidation reaction is inside the pit, while the reduction reaction occurs on the surface of the steel. The corrosion pit penetrates from the steel surface and extends vertically downward the steel.

### 2.2. Material Properties of Tested Q235 Coupon Specimens

In this paper, the tested specimens adopted Q235 low-carbon steel. The chemical composition of Q235 steel is presented in Table 1.

**Figure 2:**
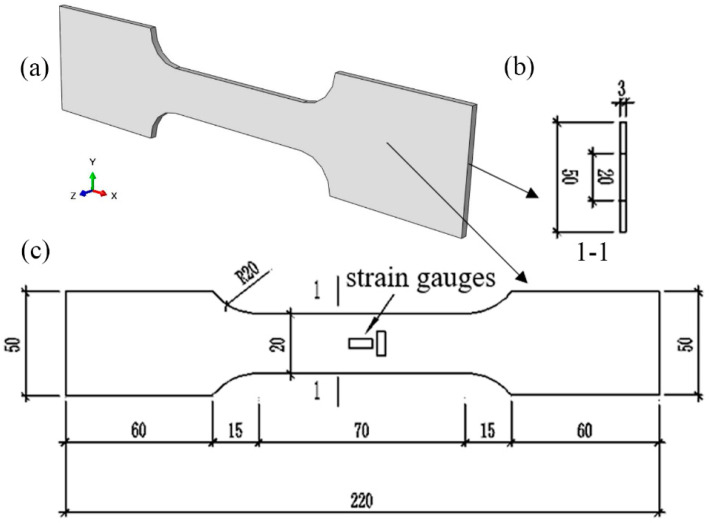
(**a**) 3D model, (**b**,**c**) Diagram and dimension of a standard coupon test specimen.

Mechanical properties of the Q235 steel are measured by the tensile coupon test according to the standard of GB/T228.1-2010 [32]. The dimensions of tensile coupon test specimens are presented in Figure 2.

### 2.3. Sulfate Corrosion Test

The steel standard tensile coupon test specimens are corroded through electro-corrosion. The steel specimen is set as the anode while the steel electrode is set as the cathode. In this process, electrons transfer between the interface of the steel-corrosion solution, which caused oxidation and reduction reactions. Such reactions are governed by Faraday’s law where the amount of electrochemical reaction caused by the flow is proportional to the amount of electricity passed, as described in Equation (1):

(1) $$m=\frac{MQ}{Fn}$$

where *M* is the reactant molecular weight; *Q* represents the electricity passed from the electrode; *m* is the mass of steel specimen under sulfate corrosion; *n* corresponds to the molar quantity of electrons when 1 mol reactant electrolysis; and *F* is the Faraday constant, which is 96,485 c·mol^−1^.

**Figure 3:**
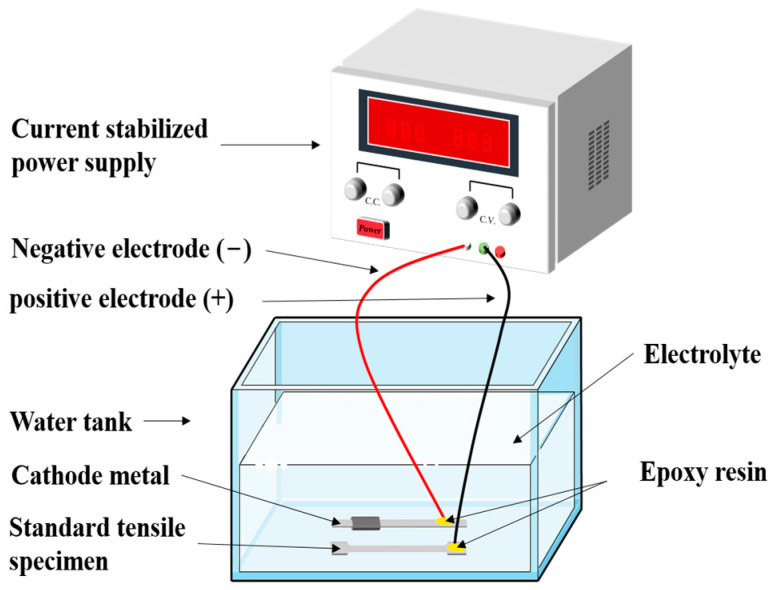
Schematic diagram of electric corrosion of steel standard tensile coupons.

The test device is presented in Figure 3, which is mainly composed of a water tank, a stabilized power supply, an artificial electrolyte, and power cords. At first, the artificial corrosion solution is poured into the water tank. Then, three steel standard tensile coupon test specimens belonging to one group are immersed into the electrolyte and placed in parallel. The height of the electrolyte is no less than the 2/3 height of the water tank, meanwhile, the wood bricks of the same size are placed under the test specimens to ensure that the specimens are fully corroded. Subsequently, the positive and negative electrode of the stabilized power supply is connected to the steel specimen and conductive rod, respectively, as shown in Figure 3. According to Faraday’s law, the electrified time is determined by Equation (2):

(2) $$\Delta T=\frac{\Delta m}{KI}$$

where Δ*m* is the loss of mass of steel specimen, g; *K* is equivalent electrochemical of Q235 steel tube, *K* = 1.024 g/A·h; *I* is the current, A (Ampere); Δ*T* represents the electrified time, h (hour). The direct current in this test is controlled as 2 × 10^−3^ A/cm^2^.

When the three specimens in one group achieved the mass of loss as requested, which corresponds to the expected corrosion rate, the power of the current stabilized power supply needs to be turned off. Then the steel specimens are removed from the corrosion solution to clear the loose corrosion products on the surface with a soft bristle brush. If the corrosion products are difficult to remove, the wire brush is adopted. Meanwhile, the steel specimens are washed with clean water to ensure that the corrosion products are completely cleared, so that the specimens would not further corrosion and ensure that the steel surface is not damaged [33].

### 2.4. Tensile Coupon Test

**Figure 4:**
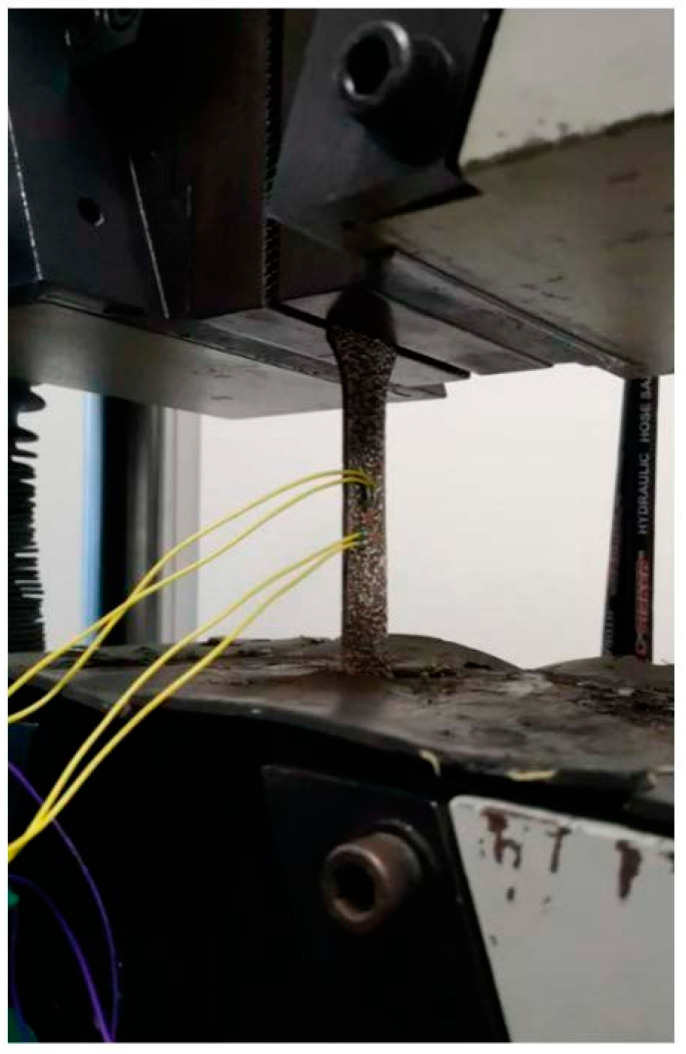
Steel coupon tensile test after sulfate corrosion layout of strain gauges.

In this paper, the resistance method is used to measure the horizontal and vertical deformation of steel standard tensile coupon test specimens. Firstly, sand the test specimen in the middle, and then degrease and clean the grinding place with alcohol. Subsequently, the strain gauges (BX120-3AA, Yiyang, China) are pasted on the location where treated in the previous step in vertical and horizontal directions. Finally, the length before stretching is measured by a Vernier caliper (DEGUQMNT, Shanghai, China). Strain control is adopted with the hydraulic actuator at a constant rate of 0.00025/s. The steel standard tensile coupon test after corrosion with steel thicknesses of 3.0 mm and 4.5 mm is presented in Figure 4.

## 3. Results and Discussion

### 3.1. Potentiogram Analysis and Gibbs Free Energy

**Figure 5:**
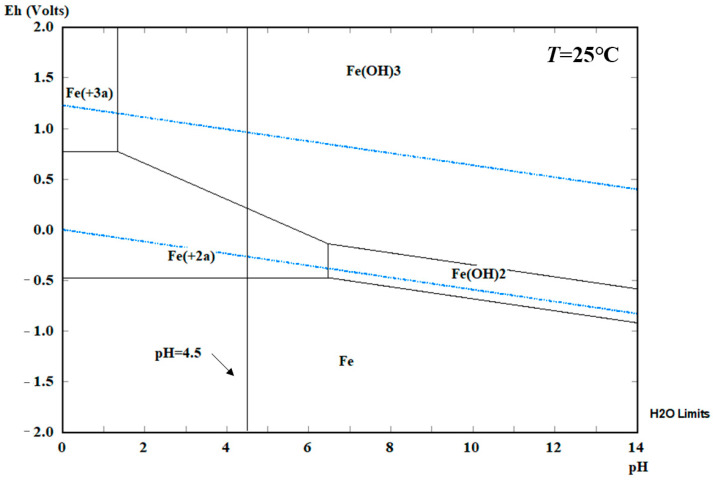
Potentiogram of steel standard tensile coupon test specimens.

Figure 5 presents the potentiogram of steel specimens at 25 °C and one atm. It can be seen that high oxidation state substances are located above low ones. Solid substances are located on the side with a higher pH value, while dissolved substances are located at a relatively low pH. Figure 5 shows the area within the two blue lines is defined as the water stability area, iron exists as Fe^2+^ and Fe^3+^, which means that steel specimens are unstable in the solution. The pH value of the electrolyte in this paper is set as 4.50, the steel specimens in this condition present corrosion. Horizontal lines in Figure 5 represent the oxygen and proton reduction of the steel corrosion. Moreover, the steel used in structural engineering contains impurities, which will corrode due to acid rain in a lower pH value without a power supply in areas Ⅰ and Ⅱ.

According to the thermodynamic principle, the direction and limits of the corrosive reaction of steel specimens can be distinguished by the change of Gibbs free energy (*G*), as expressed in Equation (3).

(3) G=U−TS+pV=H−TS

In the process of corrosive battery reaction of a metal, combined with Faraday’s law, Gibbs free energy could be described as Equation (4):

(4) $${\left(\Delta G\right)}_{T.P}=-nF\eta$$

where the unit of Δ*G* is (kJ/mol), *n* denotes the stoichiometric number of electrons consumed in the electrode reaction; *F* denotes the Faraday constant; *η* denotes the electromotive force of a reaction (V).

**Figure 6:**
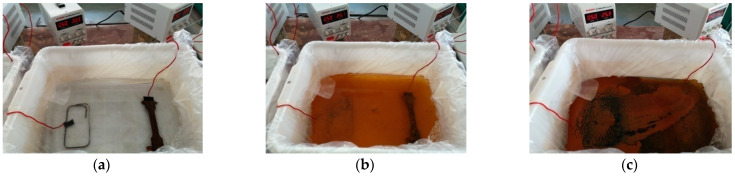

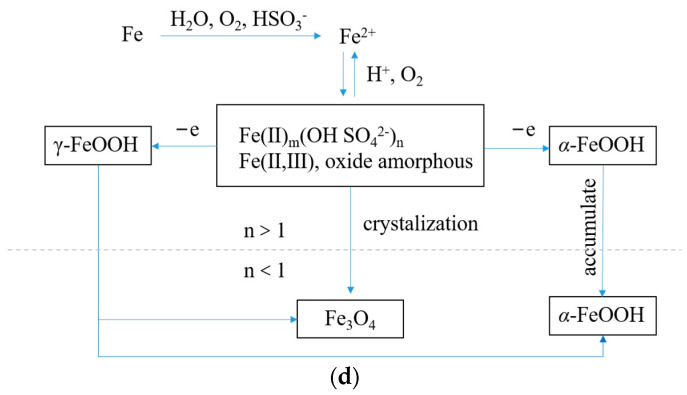
Change of electrolyte in different experimental periods (**a**) no power, (**b**) medium term, (**c**) later stage and (**d**) a corrosion flow of Q235 steel.

The initial corrosion products on the surface of Q235 steel are mainly Fe(II) and Fe(III) amorphous oxides with relatively high chemical activity, which uniformly cover the surface of steel specimens after reaching a certain cumulative amount. The formation of *γ*-FeOOH mainly comes from the crystallization reaction of amorphous components in the rust layer, while the formation of Fe_3_O_4_ may come from both the reduction and crystallization of amorphous components and the transformation of *γ*-FeOOH crystal form, as shown in Figure 6.

### 3.2. Open Circuit Potential and Linear Polarization Curve

**Figure 7:**
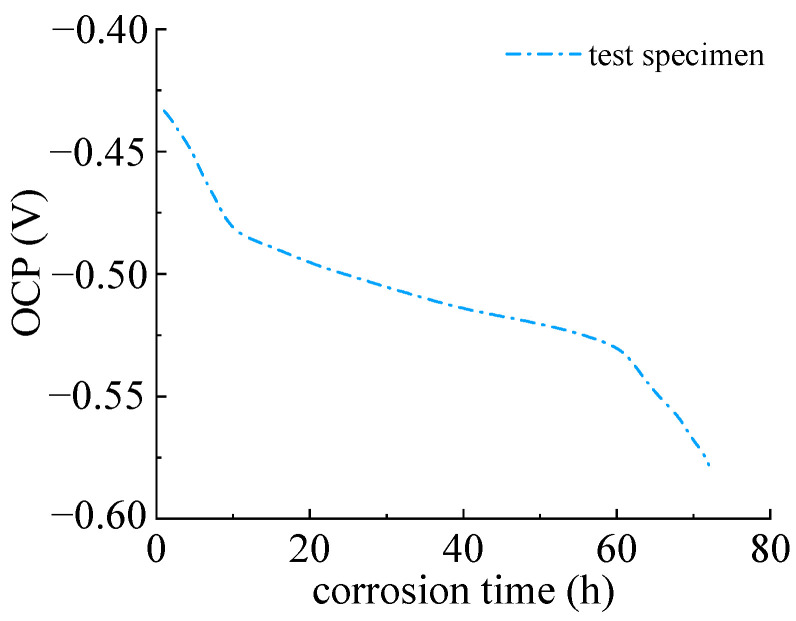
Open circuit potential of Q235 steel specimen.

Figure 7 depicts the open circuit potential of the Q235 steel specimen within 72 h. It can be seen that open circuit potential decreased rapidly at the beginning. This is because of the initial corroded layer, which provides a formed passivation film on the surface of the steel specimen. The electrolyte solution and oxygen have not yet been reached. The corrosion potential remained at a relatively high value. The initial open circuit potential was −0.429 V and decreased to −0.578 V at 72 h. This indicated that the sulfate corrosion of Q235 steel started, the electrolyte solution and oxygen diffusion into the metal interface. The anodic reaction of steel accelerated with the decreasing of corrosion potential due to the large amount of porosity of corrosion products, which caused further electrochemical reactions.

**Figure 8:**
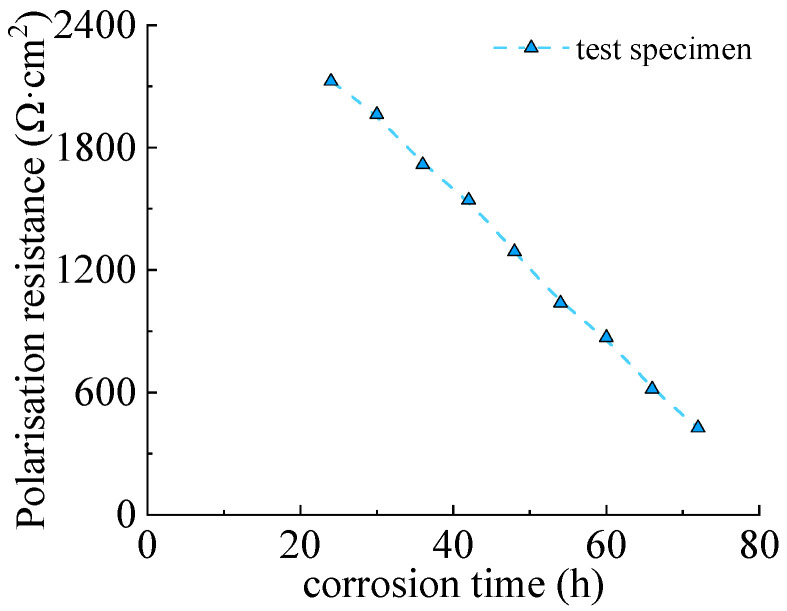
Polarization resistance of Q235 steel specimen.

The polarization resistance curve of Q235 steel was presented in Figure 8. It can be seen that the polarization resistance decreased with the corrosion time, which illustrated that the corrosion was accelerated and the corroded area enlarged. The tendency of descending was obvious within the 72 h due to the fact that the corrosion products with limited impermeability since they are loose [34].

### 3.3. SEM Analysis

The standard tensile coupon test of steel specimens subjected to sulfate corrosion indicates that the mechanical properties of the steel were uniformly reduced with the corrosion rate. The pits on the steel specimens are distributed randomly, and the size and form of the pits were different with different corrosion rates. Thus, the effect of corrosion rate on the microscopic properties of steel is analyzed by SEM.

In this section, the steel specimens with a thickness of 3.0 mm are analyzed as typical examples. The steel specimens after the tensile coupon test are cut into small slices below the size of 2 mm × 2 mm. It should be noted that the small slices for SEM are cut at the location where 25 mm away from the fracture on the longer side. Then SEM specimens are sanded with sandpaper, and the SEM specimens are dried with a blower. The electron microscope used was Evo18 (Zeiss, Oberkochen, Germany).

#### 3.3.1. Surface Morphology Analysis

**Figure 9:**
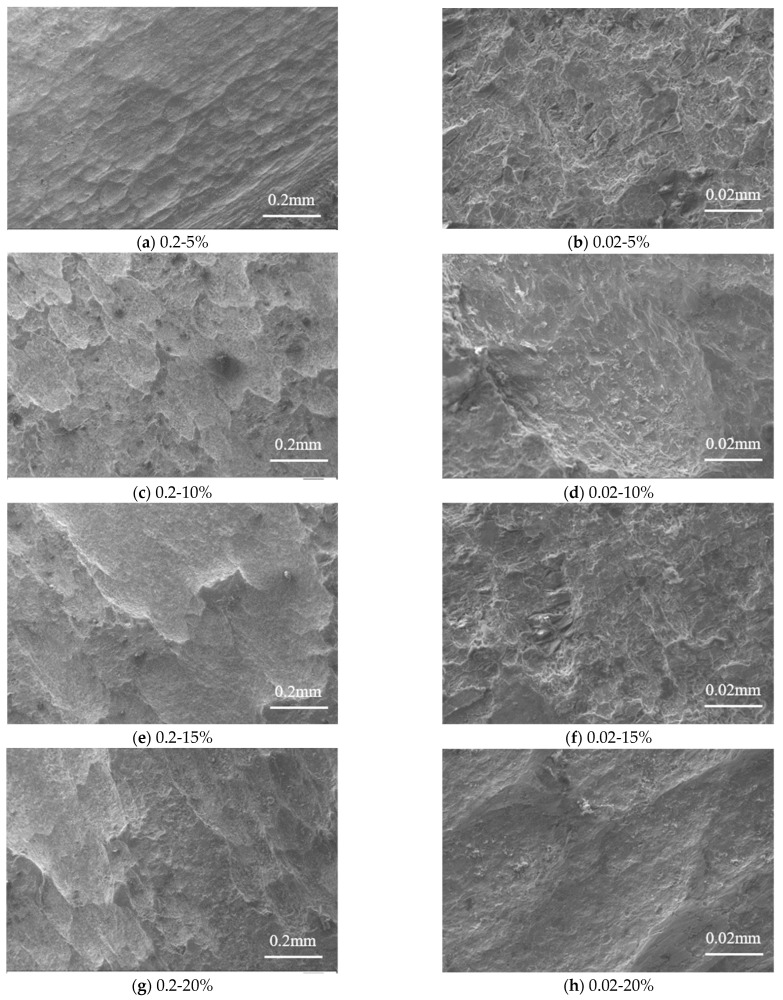

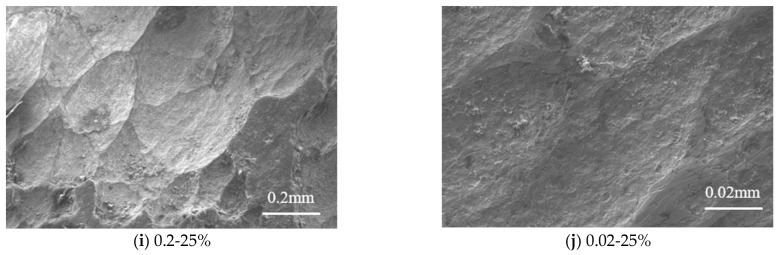
Micromorphology of standard tensile coupon steel with different sulfate corrosion rates at the thickness of 3.0 mm. Note: 0%, 5%, 10%, 15%, 20%, and 25% strands for the corrosion rate.

Figure 9 presents the microstructure of steel specimens at various magnifications. The comparison between these microstructures can observe that the corroded Q235 steel presented the characteristic of pitting corrosion. It can be seen that the corrosion pits were shallow quasi-circular shapes when the corrosion is 5%. Then the corrosion pits grew laterally, and the surface area of the corrosion pits increased but the numbers decreased. As the corrosion rate increased, the smaller corrosion pits merged together and became broad depressions, which presented characteristics of uniform corrosion. As the corrosion rate further increased, the characteristic of pitting corrosion occurred again, which indicated that the corrosion developed along the thickness direction of the Q235 steel. When the corrosion rate achieved 25%, the distribution range of corrosion depth increased. With the continuous increase of corrosion products, the corrode reaction gradually stops at the surface of steel due to the corroded layer causing difficulties in oxygen absorption and hydrogen evolution reaction. The corrosion in the direction of thickness is no longer developed. However, if the pH value is relatively lower, then the corrosion pits will develop on the surface of steel and form corrosion pits with larger diameters, which causes the connection of adjacent pits to become larger pits. New corrosion pits would occur and develop on those larger pits.

#### 3.3.2. Tensile Fracture Morphology

**Figure 10:**
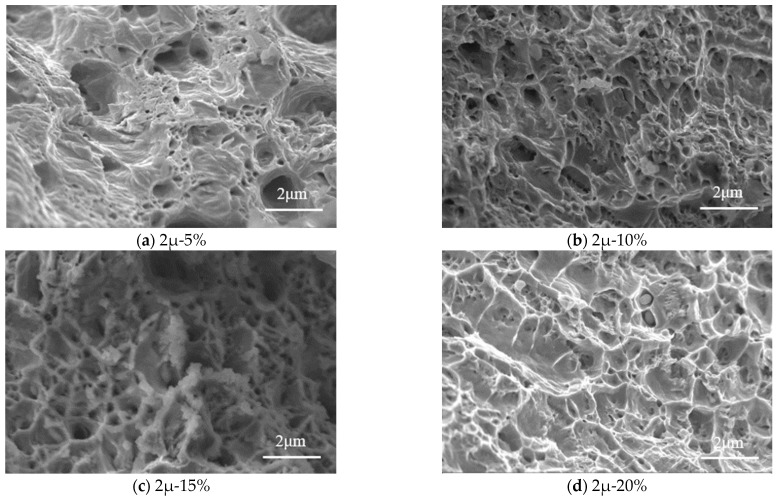

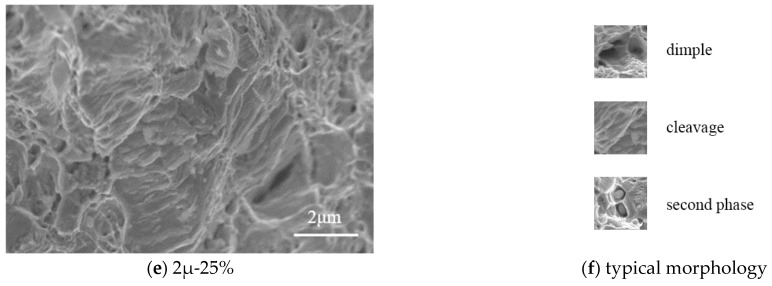
Fracture micromorphology of standard tensile coupon steel with different sulfate corrosion rates at the thickness of 3.0 mm. Note: 0%, 5%, 10%, 15%, 20%, and 25% strands for the corrosion rate.

Figure 10 shows the microstructures of tensile specimens where the tensile fracture occurred. The specimens with a thickness of 3.0 mm are analyzed under the magnification of 5000. The corrosion rate ranges from 5% to 25%. It can be seen that the microstructure of the steel can be separated into two categories: (1) intercrystalline fracture, and (2) cleavage fracture.

For the corrosion rate *γ* ≤ 15%, the steel shows an intercrystalline fracture, where a dimple was obvious. The number of dimples decreases with the increasing corrosion rate. Meanwhile, dimples vary from large and deep to small and shallow. It indicates that the deformability of steel becomes weaker with the increasing corrosion rate. When the corrosion rate increases to 20%, the microstructure of dimples and cleavage planes exist simultaneously. It indicates that the steel varies from ductile to brittle, and the brittle failure mode is obvious. The brittle cleavage fracture of metallic materials without plastic deformation and cleavage is caused by dislocation. The ductile becomes weaker due to the non-uniform stress concentrations. When the corrosion rate increases to 25%, the cleavage planes gradually increase but the dimples disappear. It illustrates that the steel occurs brittle failure. According to the test results of tensile coupons (Table A1), although the elongation of the tensile coupon specimens decreases with corrosion rate, all the elongation exceeds 5%. Thus, steel specimens are ductile and subjected to sulfate corrosion. The transition from ductile to brittle in microstructure is mainly due to the non-uniform stress distribution.

Another reason for this change is hydrogen embrittlement (HE), which caused the decrease in tensile strength and elongation. Acid rain provides a source of hydrogen, as a kind of dilute sulfuric acid solution. According to Figure 10a–e, the fracture of the steel varied from ductile fracture to brittle fracture, which was the major characteristic of HE. This transformation is due to the transgranular to intergranular fracture which is caused by hydrogen. The high-stress concentration at the tip of the pit constitutes the most favorable condition for crack initiation. The cleavage fractures are associated with locally high strains due to the high dislocation densities beneath fracture surfaces.

The deviation of the surface of the corroded steel specimen compared to its non-corroded counterparts can be considered as a stochastic process *Z*(*x*), which has self-similarity and self-affine. Therefore, fracture theory can be introduced to characterize the surface of corroded components.

The depth of corrosion pits roughly follows the normal distribution.

(5) $$\bar{d}=\left|\bar{Z}\right|=\frac{1}{MN}\sum_{i=1}^{M}\sum_{j=1}^{N}\left|Z\left(x_{i},y_{i}\right)\right|$$

(6) $${\bar{d}}_{\mathrm{sd}}=\sqrt{\frac{1}{MN}\left(\sum_{i=1}^{M}\sum_{j=1}^{N}{\left(Z\left(x_{i},y_{i}\right)-\bar{Z}\right)}^{2}\right)}$$

where *M* and *N* is the number of scanning points; $Z\left(x_{i},y_{i}\right)$ is the coordinate of the scanning point; $\bar{Z}$ is the mean value of scanning point.

Power spectrum analysis can be used to study the surface features of corroded steel. Unitized $Z\left(x_{i},y_{i}\right)$ is used as the sample function of *ζ*(*x*, *y*), *ζ*(*x*, *y*) = *Z*(*x*, *y*) + ${\bar{d}}_{\mathrm{sd}}$. Discrete two-dimensional power spectral density of bilateral corrosion depth can be described as

(7) $$S\left(\omega_{1},\omega_{2}\right)={\bar{d}}_{\mathrm{sd}}^{2}\frac{a^{2}}{4\pi }\exp\left[-{\left(b\omega_{1}\right)}^{2}-{\left(b\omega_{2}\right)}^{2}\right]$$

where *a* and *b* is the parameter corresponding to corrosion rate.

Corrosion pits varies with the increasing corrosion rate, which presented obvious cyclic process.

**Figure 11:**
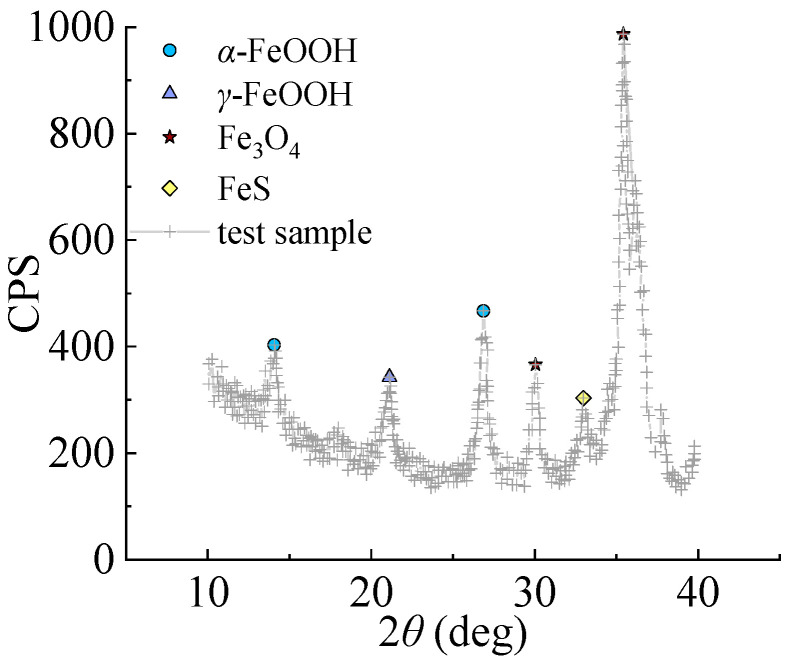
XRD pattern of sulfate corrosion products.

Figure 11 shows XRD of Q235 low carbon steel under sulfate corrosion. It can be seen that the final products mainly include *α*-FeOOH, *γ*-FeOOH, and Fe_3_O_4_. The total corrosion included the following process:

(1) Electrochemical corrosion begins:

$\mathrm{Anode}:\;\mathrm{Fe}\to {\mathrm{Fe}}^{2+}\left(\mathrm{aq}\right)+2e^{-}$
$\mathrm{Cathod}:\;O_{2}+4H^{+}+4e\to 2H_{2}O$

(2) Formation of FeOOH:

${\mathrm{HSO}}_{3}+\frac{1}{2}O_{2}\to {\mathrm{SO}}_{4}^{2-}+H^{+}\left(\mathrm{aq}\right)$
${\mathrm{Fe}}^{2+}+{\mathrm{SO}}_{4}^{2-}+xH_{2}O\to {\mathrm{FeSO}}_{4}\cdot xH_{2}O$
${4\mathrm{FeSO}}_{4}\cdot xH_{2}{O+O}_{2}+\left(6-x\right)H_{2}O\to 4\mathrm{FeOOH}+4H_{2}{\mathrm{SO}}_{4}$
$2\mathrm{FeOOH}\to \gamma {-\mathrm{Fe}}_{2}O_{3}+4H_{2}O$

(3) Formation of Fe_3_O_4_:

$3\mathrm{FeOOH}+\frac{1}{2}O_{2}+3H^{+}\to {\mathrm{Fe}}_{3}O_{4}+3H_{2}O$

### 3.4. Failure Patterns of Specimens

It can be seen that the failure patterns of steel standard tensile coupon test specimens include two modes: (1) normal fault (the fracture is perpendicular to the long axis of the specimen, *φ* = 90°); (2) and oblique fault (the fracture intersects with the long axis, *φ* ≠ 90°).

**Figure 12:**
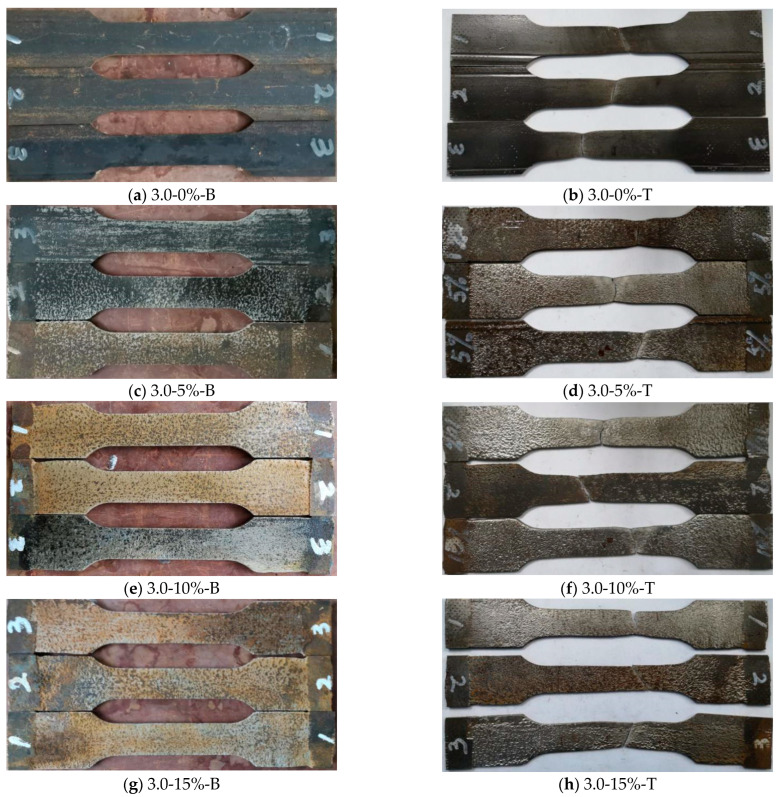

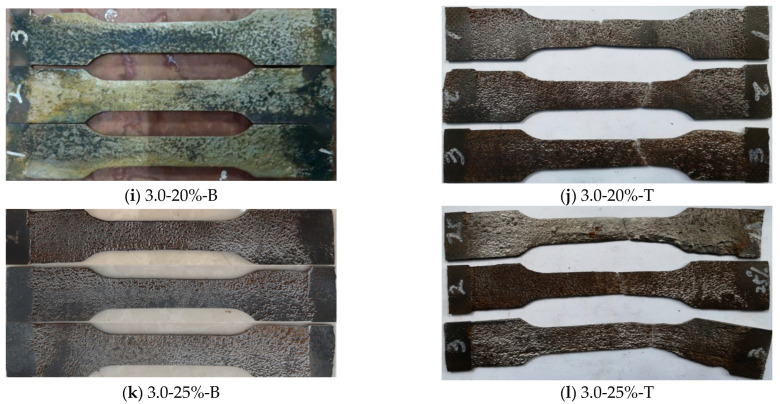
Comparison of standard coupon tensile test specimens with different corrosion rates before and after failure at a thickness of 3.0 mm. Note: 3.0-0%-B/T represents the steel specimen with a thickness of 3.0 mm, the corrosion rate is 0%, and B stands for steel specimen before tensile coupon test, while T stands for the steel specimen after the test.

**Figure 13:**
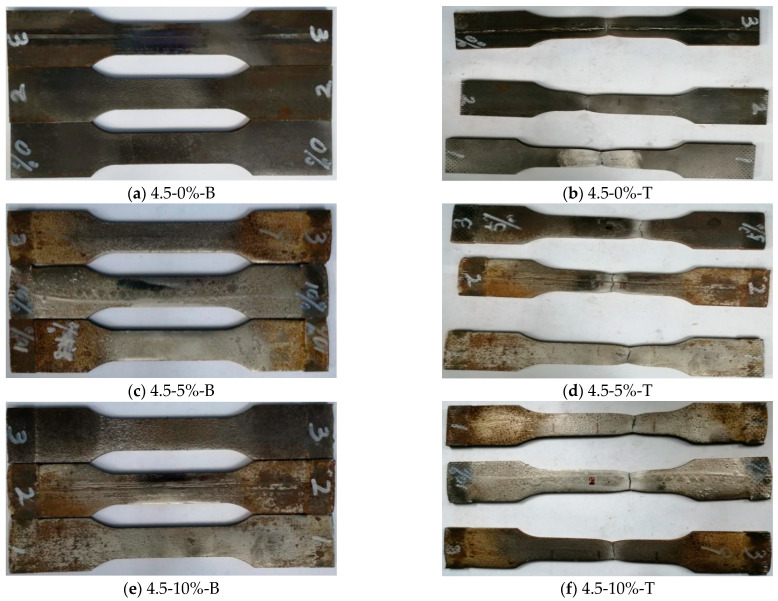

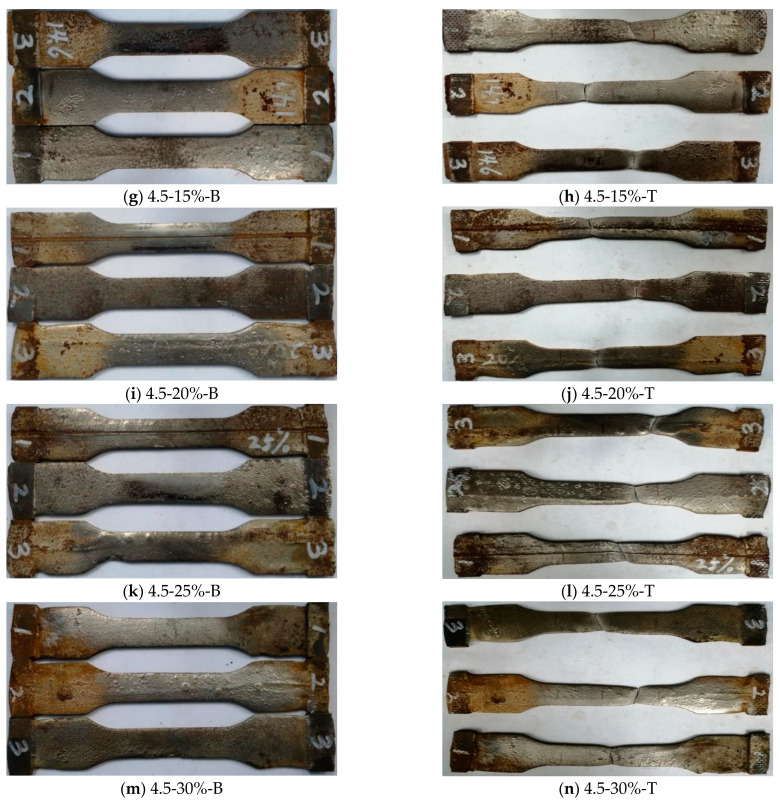
Comparison of standard coupon tensile test specimens with different corrosion rates before and after failure at a thickness of 4.5 mm. Note: 4.5-0%-B/T represents the steel specimen with a thickness of 4.5 mm, the corrosion rate is 0%, and B stands for steel specimen before the tensile coupon test, while T stands for the steel specimen after the test.

Figure 12 presents the failure mode of the steel standard tensile coupon specimens with a thickness of 3.0 mm. If the corrosion rate is within 10%, the fracture of the specimen presented a normal fault; while if the corrosion rate ranges from 15% to 25%, the fracture of the specimen will be the oblique fault. Figure 13 shows the failure patterns of steel standard tensile coupon test specimens with a thickness of 4.5 mm. It can be observed that the two types of failure modes exist and are influenced by the corrosion rate. The corrosion rate below 20% leads to a normal fault while the corrosion rate between 20% and 30% shows an oblique fault.

The failure patterns of the test specimen show that the thickness of the steel and corrosion rate affected the corrosion resistance. With the increase of the wall thickness of steel, the oblique fault occurs at a higher corrosion rate. With the increasing corrosion rate, the oblique fault occurs in the range of the high corrosion rate for all test specimens with thicknesses of 3.0 mm and 4.5 mm, respectively. It indicates that steel with a larger thickness would delay the influence of corrosion.

It can be seen that the size of the pits is different after sulfate corrosion, and the number, size, and distribution of the pits are random. When the corrosion rate *γ* ≤ 10%, the number of pits increases with the corrosion rate, and the distribution of the pits became denser which caused the pits larger and deeper. When the corrosion rate is 15% ≤ *γ* ≤ 25%, the corrosion pits became clear and denser, and the corrosion pits are assumed as hemispherical [35].

**Figure 14:**
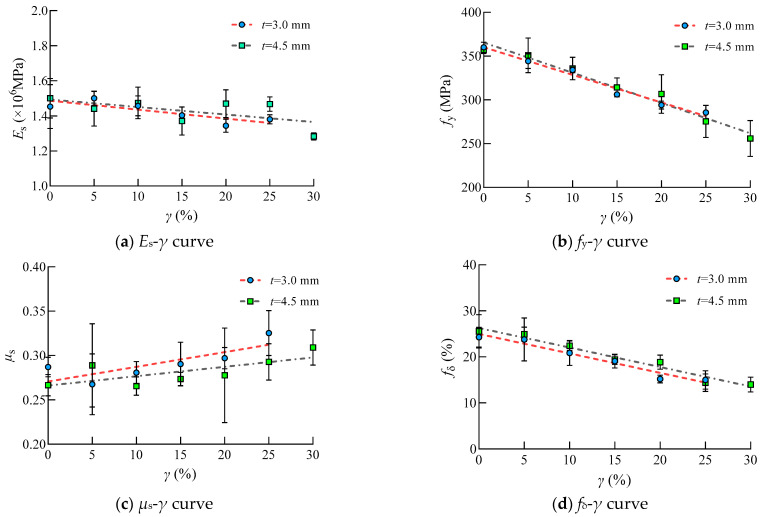
Relationships between mechanical properties of steel and corrosion rate.

The test results of steel tensile coupon specimens with thicknesses of 3.0 mm and 4.5 mm are shown in Appendix A. The following rules are utilized to distinguish the specimen: (1) The first character “T” represents the tensile coupon specimen; (2) The second Arabic number represents the thickness of the steel; (3) The third Arabic number represents the corrosion rate; (4) and the last character “a (b)” represents the different specimen in the same group. The test results of tensile coupon specimens are listed in Figure 14.

It can be seen that the value of the experimental elastic modulus, yield strength and elongation of Q235 steel decreased with the increasing sulfate corrosion rate despite the scatter of these test results. This was due to the pitting corrosion which caused the discontinuous in physical performance and local stress concentration of steel. However, the Poisson’s ratio of Q235 increased with the increasing sulfate corrosion rate, which indicated that steel changed from a plastic state to a brittle state.

It is obvious that Poisson’s ratio increased with the increasing corrosion rate. The value of Poisson’s ratio varies in the same group with relative higher SD, which is mainly due to the random distribution of corrosion pits. The increasing of Poisson’s ratio indicated that the degree of lateral shrinkage increases, and the deformation ability of the Q235 steel correspondingly decreases.

**Figure 15:**
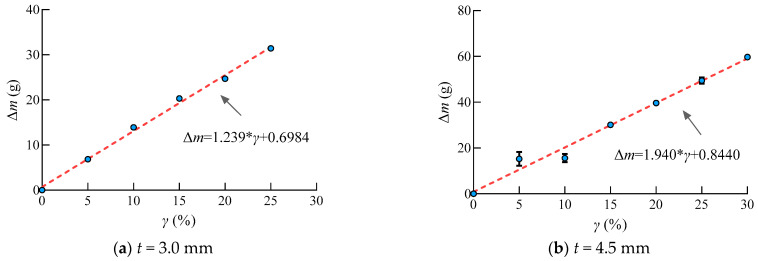
Δ*m*-*γ* relationship curve.

In this study, steel used in steel structures corroded at a rate of 0.06 mm/a. The corrosion rates 0%, 5%, 10%, 15%, 20%, and 25% correspond to the duration of 0, 2.5, 5, 7.5, 10, 12.5, and 15 years for the steel specimens with a thickness of 3.0 mm. The corrosion rates 0%, 5%, 10%, 15%, 20%, 25%, and 30% correspond to the duration of 0, 3.75, 7.5, 11.25, 15, 18.75, and 22.5 years for the steel specimens with the thickness of 4.5 mm. It can be seen that the Q235 steel weight loss with the increasing corrosion rate in Figure 15. The R square of the two linear regress formula is 0.9953 and 0.9793, respectively. However, it can be observed that the corrosion speed increased rapidly in the initial stage when the corrosion rate achieved 5%, corresponding to 2.5 years and 3.75 years for the 3.0 and 4.5 mm steel, respectively, when the weight loss was the most serious. This is because the rust layer avoided the sulfate corrosion, which caused the decrease in the corrosion rate in the later corrosion stage. This regulation matched the SEM analysis.

The mechanical properties of steel subjected to sulfate corrosion changed linearly with the corrosion rate. The quantitative relationships between these mechanical parameters and corrosion rate are as follows, within the errors of ±15%.

(8) $$f_{y,\mathrm{sc}}=\left(1-0.908\gamma \right)f_{y}$$

(9) $$E_{s,\mathrm{sc}}=\left(1-0.525\gamma \right)E_{s}$$

(10) $$\mu_{s,\mathrm{sc}}=\left(1-0.221\gamma \right)\mu_{s}$$

(11) $$f_{\delta ,\mathrm{sc}}=\left(1-1.685\gamma \right)f_{\delta }$$

**Figure 16:**
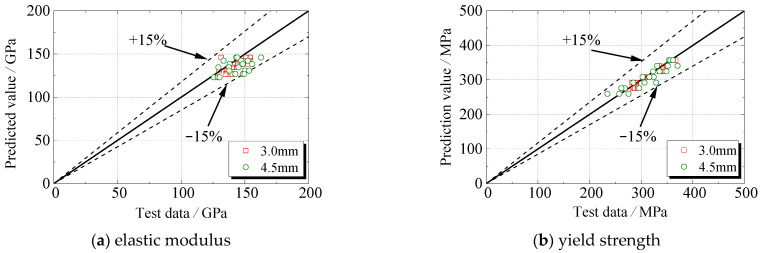

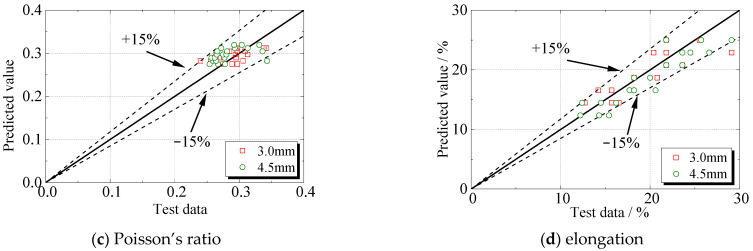
Comparison of experimental and calculated values of mechanical properties of corroded steel.

Figure 16 presented the accuracy and applicability of the model in predicting the degradation of steel specimens under sulfate corrosion. The deviation is within ±15%.

It can be seen that the elastic modulus of steel coupon specimens varies between the same group. Although the elastic modulus is not sensitive to the metallographic microstructure of steel, the test results are scattered to some degree. This is because the concentration stress occurred at the randomly distributed corrosion pits, which influenced the elastic modulus of steel.

### 3.5. Stress-Strain Curves

**Figure 17:**
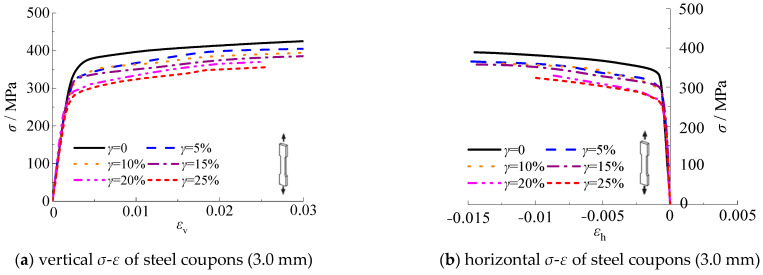

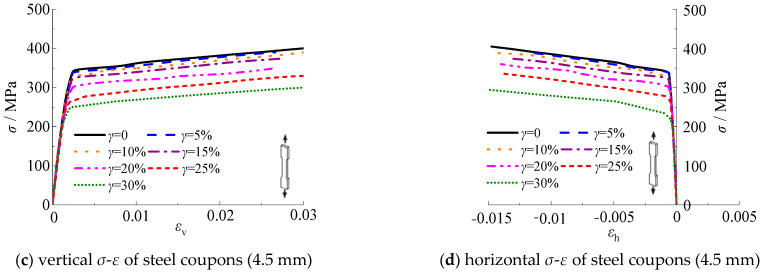
Stress-strain curves of standard steel tensile coupon test specimen.

Figure 17 shows the relationship of the longitudinal and transverse stress-strain for test specimens with thicknesses of 3.0 mm and 4.5 mm, respectively. The stress-strain curves are measured until the test specimens are fractured. It should be noted that the stress-strain curves are the average value of the three test specimens per group. It can be seen that the stress-strain curves of steel test specimens with thicknesses of 3.0 mm and 4.5 mm are similar. As shown in Figure 17, the yield strength of steel decreased with the increasing corrosion rate. The slope corresponding to the elastic-plastic stage decreased with the increasing corrosion rate, which indicates that the strength and deformation ability of the specimen decreased. 

### 3.6. Parameters of Reduce

#### 3.6.1. Ultimate Tensile Strength Reduction Factor

Ahmmad et al. [36] suggested that the ultimate strength reduction factor is a function of damage to the steel plate under corrosion. The equation of the ultimate strength reduction factor *R*_u_ is as follows:

(12) $$R_{u}=\frac{\sigma_{\mathrm{up}}}{\sigma_{u0}}$$

where *σ*_up_ and *σ*_u0_ are the ultimate tensile strengths of steel with different sulfate corrosion rates and steel with a sulfate corrosion rate of 0%.

**Figure 18:**
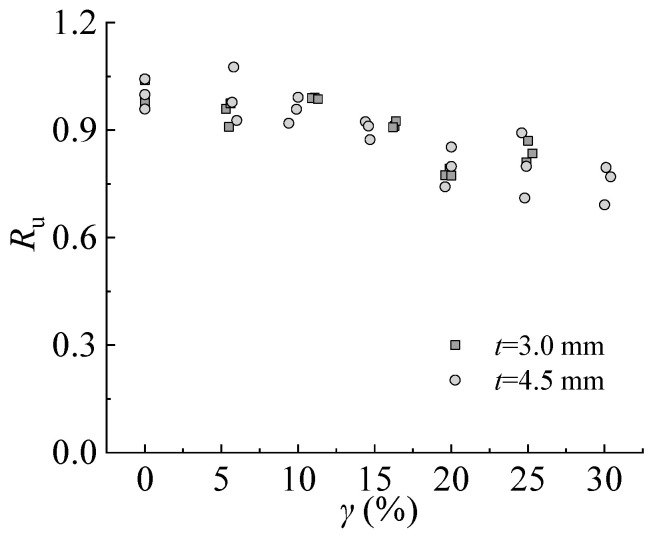
Ultimate tensile strength reduction factor.

Figure 18 shows that the strength reduction factor decreases linearly with the increasing corrosion rate from 0% to 30%. *R*_u_ decreases 13.0–19.0% when the corrosion rate increases from 0% to 25% of the steel specimen with a thickness of 3.0 mm. For the steel specimen with a thickness of 4.5 mm, *R*_u_ decreases by 10.8–28.9% and 20.5–30.8% corresponding to the corrosion rates of 25% and 30%, respectively. The effect of sulfate corrosion is more significant on steel with larger thicknesses.

#### 3.6.2. Deformability Reduction Factor

The deformability reduction factor *R*_d_ is defined as Equation (13):

(13) $$R_{d}=\frac{\delta_{p}}{\delta_{0}}$$

where *δ*_p_ and *δ*_0_ are the total elongation of steel with different sulfate corrosion rates and steel with a sulfate corrosion rate of 0%.

**Figure 19:**
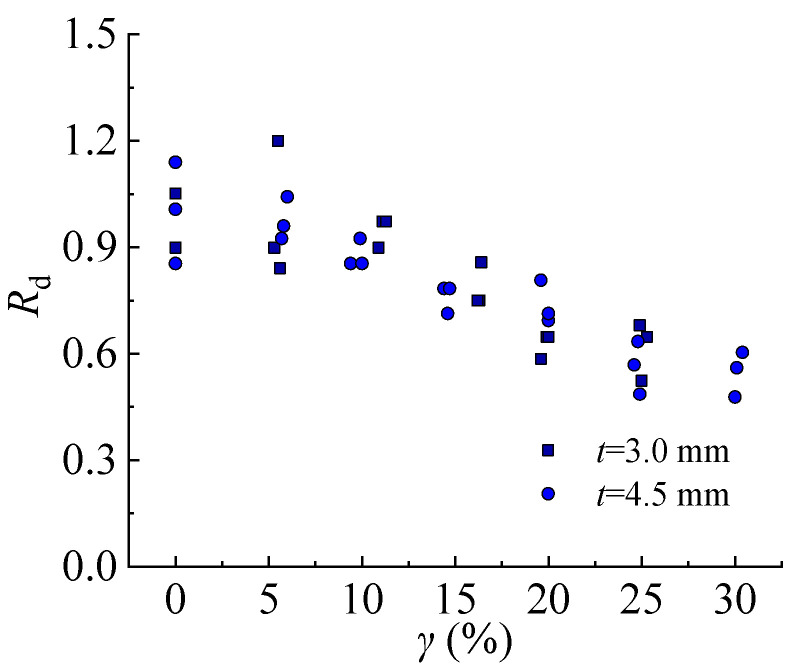
Deformability reduction factor.

The deformability reduction factor of steel decreases with the increasing corrosion rate as shown in Figure 19. *R*_d_ decreases 32–47.7% of the steel specimens with a thickness of 3.0 mm when the corrosion rate increases from 0% to 25%. Moreover, *R*_d_ decreases by 36.6–51.4% and 39.7–52.2% of the steel specimens with a thickness of 4.5 mm corresponding to the corrosion rates of 25% and 30%, respectively. It can be seen that the steel specimen with a larger thickness is more sensitive to the impact of sulfate corrosion on deformability.

#### 3.6.3. Energy Absorption Reduction Factor

The energy absorption reduction factor is introduced as Equation (14):

(14) $$R_{e}=\frac{E_{\mathrm{np}}}{E_{n0}}$$

where *E*_np_ and *E*_n0_ are the total energy absorbed by steel with different sulfate corrosion rates and steel with a sulfate corrosion rate of 0%.

**Figure 20:**
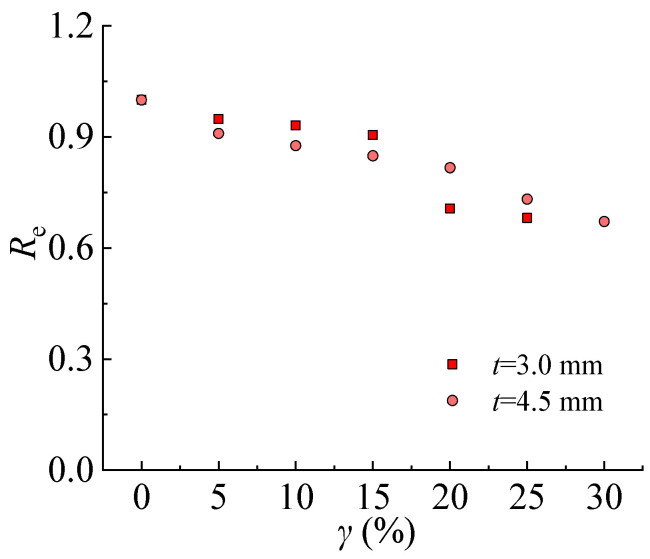
Energy absorption reduction factor.

The energy in this section is measured by integrating the area under the nominal stress-strain curves of the steel tensile coupon test. The energy absorption reduction factor decreases with increasing the corrosion rate, as shown in Figure 20. For steel specimens with a thickness of 3.0 mm, *R*_e_ decreased by 31.9% corresponding to a corrosion rate of 25%. For steel specimens with a thickness of 4.5 mm, *R*_e_ decreased by 26.8% and 32.8% corresponding to the corrosion rate of 25% and 30%, respectively. It indicates that the fracture toughness of the steel specimens decreases with the sulfate corrosion. The steel is more likely to be damaged under a high corrosion rate, i.e., a longer time subjected to sulfate corrosion or a higher concentration of corrosion solution. For the steel specimen with a larger thickness, it is harder for fracture damage to occur fracture.

The elongation of the steel specimen deteriorates severely as the corrosion rate increases, and the decrease in ductility is detrimental to the seismic performance of the steel structure. When experiencing a major earthquake, steel structures are subjected to significant cyclic loads, and materials enter the plastic stage, dissipating seismic energy in the structure. At this point, there is a problem of fatigue failure at the material level [37,38].

#### 3.6.4. Empirical Design Method

It can be seen that the reduction factors of deformability and energy absorption capacity are reduced by about 50% and 33% with the increasing corrosion rate increasing from 0% to 30%, respectively, while the ultimate tensile strength of steel is reduced moderately. It can be found that all the reduction parameters presented discreteness, which is due to the random distribution of the pits. However, the reduction factors of *R*_u_, *R*_d_, and *R*_e_ decreased linearly with the increasing corrosion rate. According to the least square method, reduction factor *R*_u_, *R*_d_, and *R*_e_ is described as follows:

*R*_u_ = −0.0087*γ* + 1.020 (15)

*R*_d_ = −0.0172*γ* + 1.042 (16)

*R*_e_ = −0.0113*γ* + 1.005 (17)

where *γ* is the corrosion rate, ranging from 0% to 30%.

In acid rain environments, the FeOOH and Fe_3_O_4_ are formed by crystallization, since the *γ*-FeOOH is mainly formed by amorphous oxides, which can be further converted into *α*-FeOOH and revert to Fe_3_O_4_. The rust layer fall off with the increasing corrosion rate, which caused the corrosion pits to develop along the thickness direction. The stress of the specimen became non-uniform. When the main stress bypassed the corrosion pit, it bent and generated a three-dimensional tensile stress; the more severe the stress concentration, the more brittle the steel tends to be. In the steel with larger thickness, the three-dimension tensile stress was greater at the corrosion pit, which was due to the significant limitation of the shrinkage deformation in the steel thickness direction at the center of the corrosion pit.

### 3.7. Finite Element Analysis of Axially Loaded Sulfate Corrosion CFST Stub Columns

**Figure 21:**
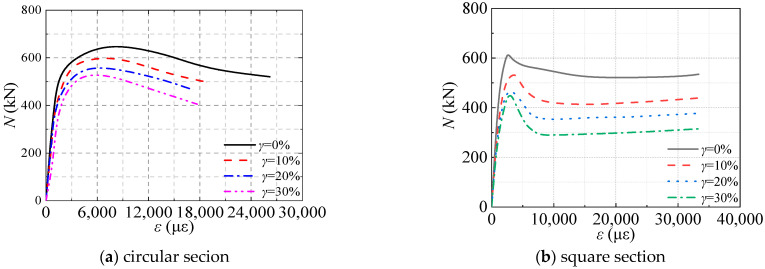
Axial load (*N*)-axial strain (*ε*) curve.

The detail of finite element models was presented in ref. [5]. Figure 21 presented the axial load (*N*)-axial strain (*ε*) curve for circular and square section CFST stub columns corresponding to sulfate corrosion rates of 0%, 10%, 20%, and 30%. It can be seen that the *N*-*ε* curve includes the elastic stage, elastic-plastic stage, peak load, and descending stage. The elastic limit point of the CFST stub column subjected to sulfate corrosion corresponds to the smaller strain. The elastic modulus of the specimen decreased with the increasing corrosion rate. In the elastic-plastic stage, the ultimate strain corresponding to peak load decreased with the corrosion rate as well as the peak load. In the descending stage, the bearing capacity of the CFST column decreased. Compared with the reference specimen of *γ* = 0%, the specimens subjected to sulfate corrosion would end the descent stage with a smaller strain. 

**Figure 22:**
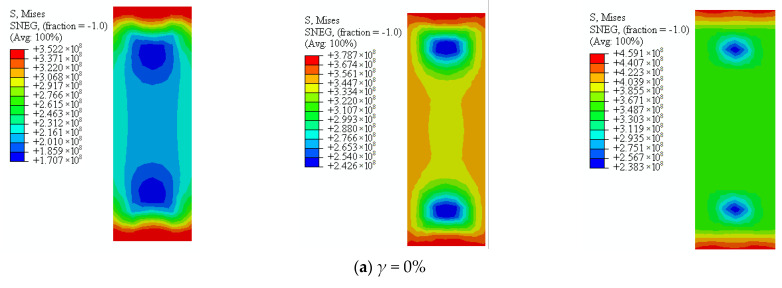

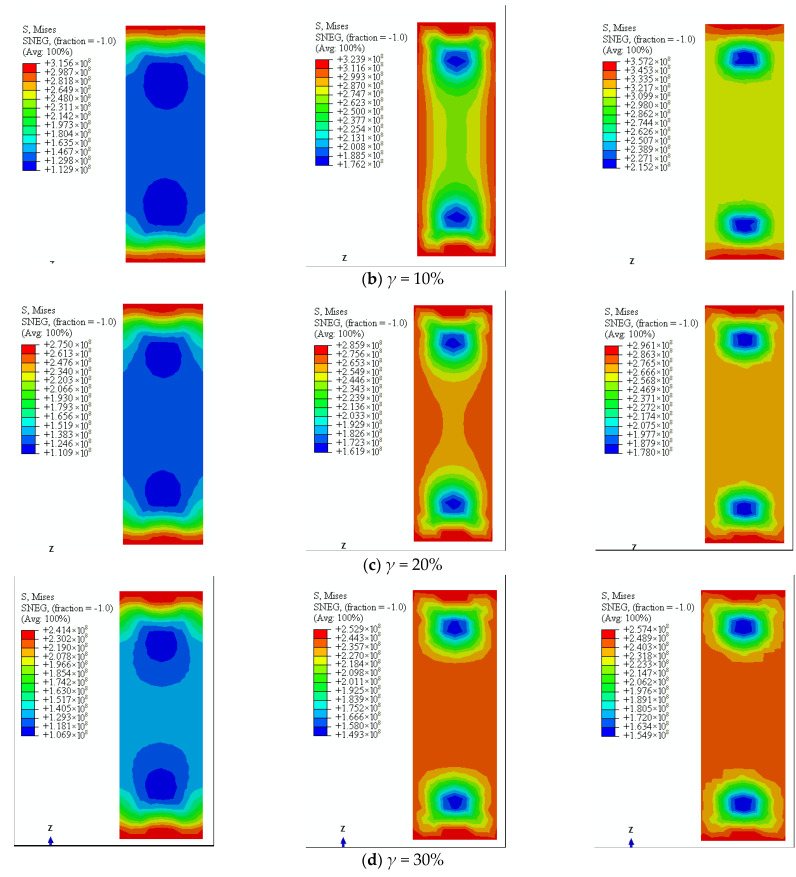
Von Mises stress of steel tube subjected to sulfate corrosion.

Von Mises strength of square sulfate corrosion steel tube with thickness of 4.5 mm is shown in Figure 22. It can be seen that the stress of the steel tube increased in the process of axial compression. The stress of steel tube decreased with the increasing sulfate corrosion rate. In the elastic stage, the stress decreased by 10.39%, 21.92%, and 31.46%. In the elastic-plastic stage, the stress of steel tube decreased by 14.47%, 24.50%, and 33.22% as the corrosion rate increased from 0% to 30%. In the descending stage, the stress of steel tube decreased by 5.68%, 21.81%, and 32.03% corresponding to the corrosion rate of 10%, 20%, and 30%. 

The ultimate compressive strength of the CFST stub columns subjected to sulfate corrosion can be described as follows:

(1) Circular section

(18) $$N_{us}=f_{\mathrm{sc}}\left(\gamma \right)A_{\mathrm{sc}}$$

(19) $$f_{\mathrm{sc}}\left(\gamma \right)=\left[1.212+B\xi \left(1-1.0075\gamma \right)+C\xi^{2}{\left(1-1.0075\gamma \right)}^{2}\right]$$

where *B* = 0.176*f*_y_/213 + 0.974; *C* = −0.104*f*_ck_/14.4 + 0.031; *N*_us_ is the ultimate compressive strength subjected to sulfate corrosion; *γ* represents corrosion rate; *ξ* denotes confinement coefficient, *ξ* = *A*_s_*f*_y,s_/*A*_c_*f*_ck_, *A*_s_ and *A*_c_ are area of steel tube and concrete core, *f*_y,s_ is the yield strength of sulfate corroded steel, and *f*_ck_ is the compressive strength of the concrete core.

(2) Square section

(20) $$N_{\mathrm{us}}=\left[1-6.25\gamma \left(0.006\xi^{2}+0.019\xi +0.082\right)\right]f_{\mathrm{sc}}A_{\mathrm{sc}}$$

where $f_{\mathrm{sc}}=\left(1.212+B\xi +C\xi^{2}\right)f_{\mathrm{ck}}$, *B* = 0.131*f*_y_/213 + 0.723, *C* = −0.070*f*_ck_/14.4 + 0.026.

**Figure 23:**
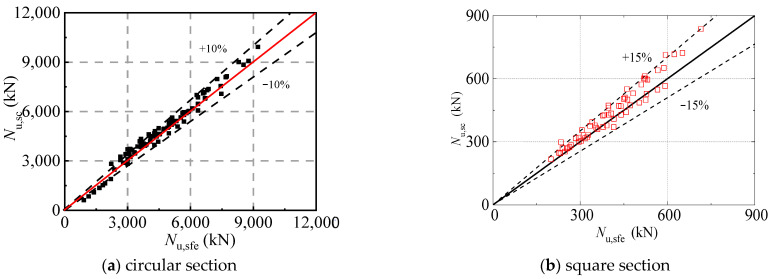
Comparison between proposed formula and finite element results.

It can be seen from Figure 23 that the proposed formula for axially loaded circular and square section CFST stub columns subjected to sulfate corrosion has high accuracy. The mean value of circular columns is 0.895 with a COV of 0.034, and the mean value of square columns is 0.937 with a COV of 0.004. It should be noted that the error interval of the square CFST columns is 15% compared to that of circular columns of 10%. This is due to the consideration of the random local buckling of the square steel tube. 

## 4. Equivalent Reduction Thickness and Verification

### 4.1. Theory Model

The corrosion of steel specimens in a corrosive environment involves complex chemical processes, and the corrosion pits are distributed randomly. During the corrosion process, corrosion pits occurred in the beginning, and then they developed and blended. After the rust layer peels off, the steel presented the characteristics of nonuniform overall corrosion. Thus, the equivalent thickness considering the depth of corrosion pit is proposed.

According to Equation (4), the Gibbs free energy of a steel tube per unit volume is defined as follows:

(21) $$\rho {\left(\Delta G\right)}_{T,P}=-m\eta$$

where *m* is the total charge of steel in the electrochemical reaction per unit volume, *m* = *nFρ*/*M*; *M* is the atomic weight of steel.

Since the pressure (*P*) and volume (*V*_s_) of the system keeps constant, the derivation of time is on both sides of Equation (4). The rate of the Gibbs free energy under isothermal conditions in the process of sulfate corrosion is as follows:

(22) $$\rho \dot{G}=-\dot{m}\eta +\rho \frac{\partial G}{\partial D}\dot{D}$$

where *D* is the damage internal variable.

(23) $$-\dot{m}\eta +\rho \frac{\partial G}{\partial D}D\leq 0$$

Equation (23) illustrates the loss of dissipation energy due to electrochemical corrosion. It can be clearly seen in Figure 9 and Figure 10 that the corrosion pit distributes on the surface of the whole specimen, and the shape of the corrosion pit is presented as a hemisphere or semi-ellipsoid [35,39]. It assumes that the density of steel keeps constant. According to Faraday laws, the volume changes of a corrosion pit with time are as follows:

(24) $$\frac{dV}{dt}=\frac{MI_{c}}{nF\rho_{s}}$$

where *M* is the atomic weight of the metal; *I*_c_ represents the current that caused the corroded pit; *n* is the number of metal ions; *F* is the Faraday constant; *ρ*_s_ is the density of steel.

In this paper, the shape of the corrosion pit is simplified as the hemisphere, thus the volume of the corrosion pit is:

(25) $$V=\zeta^{2}\cdot \frac{2}{3}\pi d^{3}$$

where *d* is the radius of the hemisphere which corresponds to the depth of the corrosion pit; *ζ* is the ratio of the semi-minor axis to the semi-major axis of the ellipsoid, *ζ* = 1.0 when the corrosion pit is a hemisphere. Thus, by integrating time, Equation (24) combined with Equation (25) can be expressed as follows.

(26) $$\frac{2}{3}\pi \zeta^{2}d^{3}=\int_{0}^{t}\frac{MI_{c}}{nF\rho_{s}}dt$$

The corrosion current *I*_c_ is considered to be constant [30], according to Equation (23), the depth of corrosion pit *d* can be described as

(27) $$d=\frac{3MI_{c}}{2\pi \zeta^{2}nF\rho_{s}}\sqrt{t}$$

where *d* is the depth of corrosion pit, and for the hemisphere pit, Equation (27) can be simplified as the following.

(28) $$d=\frac{3MI_{c}}{2\pi nF\rho_{s}}\sqrt{t}$$

According to meso-damage mechanics, the porosity of steel after sulfate corrosion can be defined as [35]

(29) $$p_{v}=\frac{V_{vo}}{V_{0}}$$

where *P*_v_ is the porosity of steel after sulfate corrosion; *V*_vo_ is the void volume of steel; *V*_0_ is the volume of the steel coupons.

The basic consumptions are described as follows:

(1) The appearance and development of pore damage are caused by corrosion;
(2) There is no pit before sulfate corrosion, i.e., *V*_void_ = 0;
(3) The density of steel keeps constant in the process of sulfate corrosion.

Therefore, the porosity of steel tube can be expressed as the following.

(30) $$p_{v}=\frac{V_{\mathrm{vo}}}{V_{0}}=\frac{m}{m_{0}}=\gamma$$

Combined Equations (4) and (30), for single sided corroded specimens, sulfate corrosion rate can be expressed as follows.

(31) $$\gamma \left(t\right)=\frac{M\int_{t_{0}}^{t}I\left(t\right)dt}{nFt_{s}\rho_{s}}$$

Simultaneously, the sulfate corrosion rate for double sided corroded specimens, sulfate corrosion rate can be described as the following.

(32) $$\gamma \left(t\right)=\frac{2M\int_{t_{0}}^{t}I\left(t\right)dt}{nFt_{s}\rho_{s}}$$

Thus, the equivalent reduced thickness considering sulfate corrosion rate can be expressed as follows.

(33) $$\Delta t_{s}=\frac{M\int_{t_{0}}^{t}I(t)dt}{nF\rho_{s}}$$

(34) $$\Delta t_{s}=\frac{2M\int_{t_{0}}^{t}I(t)dt}{nF\rho_{s}}$$

The equivalent residual thickness is defined as

(35) $$t_{s}^{\prime }=t_{s}-\Delta t_{s}$$

where *t*_s_′ is the equivalent residual thickness, and *t*_s_ is the thickness of the steel before corrosion.

### 4.2. Verification of the Porposed Model

**Figure 24:**
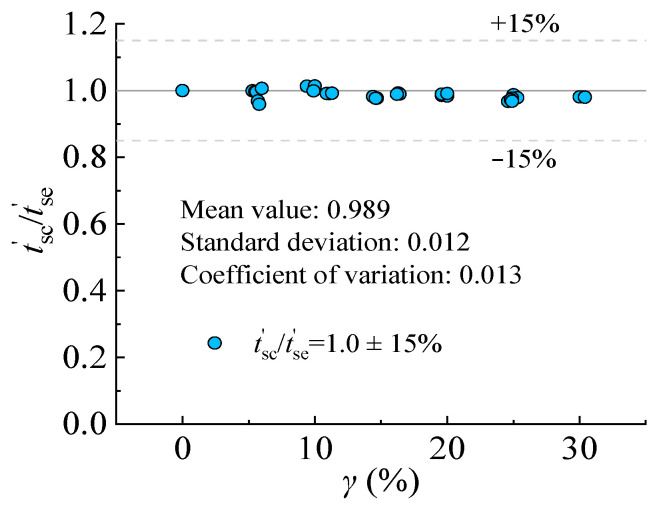
Comparison of calculated and measured equivalent thickness of standard steel coupons.

The proposed equation considering the effects of corrosion was verified through the measured value, as presented in Figure 24.

It should be noted that *t*_sc_′ is defined as the equivalent residual thickness calculated by Equation (30) and *t*_se_′ is defined as the equivalent residual thickness measured by steel coupons. The comparison results between *t*_sc_′ and *t*_se_′ is presented in Figure 18, with the average value of 0.989 and COV of 0.013.

## 5. Conclusions

Through experimental tests and theoretical analysis, the mechanical properties of steel standard tensile coupons are investigated by mechanical performance tests and SEM tests. The following conclusions can be drawn:

1. The failure mode of corroded steel standard tensile coupon includes two modes: normal fault (the fracture is perpendicular to the long axis of the specimen, *φ* = 90°), and oblique fault (the fracture intersects with the long axis, *φ* ≠ 90°).
2. For the T-3.0 specimens, the fracture of the specimen presented a normal fault and oblique fault when the corrosion rate is within 10% and ranges from 15% to 25%, respectively. For the T-4.5 specimens, the fracture of the specimen presented a normal fault and oblique fault when the corrosion rate is within 20% and ranges from 25% to 30%, respectively.
3. The strength reduction factor (*R*_u_), deformability reduction factor (*R*_d_) and energy absorption reduction factor (*R*_e_) decrease linearly with the corrosion rate from 0% to 30%. The effect of sulfate corrosion is more significant on the *R*_u_, *R*_d_, and *R*_e_ of steel with larger thicknesses.
4. The number, size, and distribution of the corroded pits are random after sulfate corrosion. When the corrosion rate is within 10%, the number of deep and dense corroded pits increases with the corrosion rate. When the corrosion rate ranges from 15% to 25%, the corroded pits became clear and denser, which generated the corroded pits of the hemisphere.
5. An equivalent thickness reduction model is proposed based on Faraday’s law and the meso-damage theory. The average ratio and standard deviation of the calculated value of equivalent residual thickness to the measured value is 0.989 and 0.012, respectively, indicating safety. The steel structures and steel-concrete composite structures may use this model to predict the remaining mechanical capacity through the reduction of steel thickness for the remaining service life.

## Figures and Tables

**Table 1:** Chemical composition of Q235 steel (mass%).

C	Si	Mn	S	P	Cu	Fe
0.20	0.22	0.59	0.027	0.018	0.02	98.925

## Data Availability

Data is unavailable due to privacy.

## Appendix A

**Table A1:** Main Parameters and Test Results of the Steel Tensile Coupons.

No.	*t*_s_ (mm)	*t*_s_′(mm)	*m*_0_ (g)	*m*_1_ (g)	*γ* (%)	*E*_s_ (GPa)	*f*_y_ (MPa)	*f*_u_ (MPa)	*μ*_s_	*f*_δ_ (%)
T-3.0-0-a	3.0	3.0	125.4	125.4	0.0	154.1	356.7	429.3	0.275	25.5
T-3.0-0-b		3.0	125.1	125.1	0.0	150.7	356.8	452.7	0.289	21.8
T-3.0-0-c		3.0	124.9	124.9	0.0	131.2	366.8	424.7	0.297	25.5
T-3.0-5-a	3.0	2.834	125.2	118.6	5.3	150.0	348.4	471.6	0.306	21.8
T-3.0-5-b		2.836	124.3	117.5	5.5	153.7	334.6	395.9	0.240	29.1
T-3.0-5-c		2.830	126.6	119.5	5.6	145.5	349.9	424.5	0.256	20.4
T-3.0-10-a	3.0	2.670	125.7	111.8	11.1	144.1	330.5	431.3	0.271	23.6
T-3.0-10-b		2.675	125.4	111.7	10.9	152.3	336.7	431.0	0.295	21.8
T-3.0-10-c		2.659	125.9	111.7	11.3	140.7	335.4	429.6	0.276	23.6
T-3.0-15-a	3.0	2.501	124.8	104.5	16.3	140.2	304.3	396.9	0.294	18.2
T-3.0-15-b		2.509	123.8	103.5	16.4	141.2	306.2	403.0	0.313	20.8
T-3.0-15-c		2.510	125.2	104.9	16.2	140.0	308.3	395.4	0.264	18.2
T-3.0-20-a	3.0	2.406	123.0	98.5	19.9	136.2	299.3	345.2	0.285	15.7
T-3.0-20-b		2.411	124.7	100.2	19.6	136.5	292.1	337.2	0.297	14.2
T-3.0-20-c		2.402	124.9	99.9	20.0	129.7	290.7	336.6	0.309	15.7
T-3.0-25-a	3.0	2.233	124.8	93.6	25.0	134.8	284.3	378.9	0.342	12.7
T-3.0-25-b		2.238	125.9	94.1	25.3	138.7	285.2	363.6	0.338	15.7
T-3.0-25-c		2.256	125.3	94.1	24.9	140.0	287.3	352.9	0.296	16.5
T-4.5-0-a	4.5	4.5	197.0	197.0	0.0	143.1	353.5	403.4	0.267	29.1
T-4.5-0-b		4.5	196.8	196.8	0.0	144.0	356.4	420.5	0.254	21.8
T-4.5-0-c		4.5	199.7	199.7	0.0	162.6	361.5	438.8	0.278	25.7
T-4.5-5-a	4.5	4.214	200.8	184.4	5.7	152.3	351.5	411.5	0.259	23.6
T-4.5-5-b		4.228	201.7	184.2	5.8	133.2	370.5	452.7	0.343	24.5
T-4.5-5-c		4.233	197.1	185.3	6.0	147.1	330.9	390.1	0.264	26.6
T-4.5-10-a	4.5	4.081	198.3	184.0	9.4	155.6	348.9	386.8	0.277	21.8
T-4.5-10-b		4.070	198.3	183.6	10.0	148.2	323.1	417.3	0.257	21.8
T-4.5-10-c		4.056	196.8	179.2	9.9	137.9	335.6	403.3	0.262	23.6
T-4.5-15-a	4.5	3.821	199.1	169.8	14.4	144.9	324.2	388.7	0.265	20.0
T-4.5-15-b		3.809	199.6	169.1	14.7	129.1	302.7	367.6	0.275	20.0
T-4.5-15-c		3.811	198.5	168.0	14.6	137.4	315.8	383.6	0.280	18.2
T-4.5-20-a	4.5	3.512	199.1	159.1	20.0	149.8	328.8	358.9	0.336	17.7
T-4.5-20-b		3.538	199.6	160.4	19.6	137.8	284.8	312.2	0.231	20.6
T-4.5-20-c		3.520	198.5	158.8	20.0	153.2	306.5	336.1	0.266	18.2
T-4.5-25-a	4.5	3.339	194.8	146.9	24.6	148.2	296.2	375.5	0.313	14.5
T-4.5-25-b		3.324	200.6	150.9	24.8	149.5	268.5	299.1	0.293	16.2
T-4.5-25-c		3.317	199.7	149.1	24.9	142.4	261.5	336.3	0.272	12.4
T-4.5-30-a	4.5	3.045	197.0	137.6	30.1	125.6	275.4	334.8	0.304	14.3
T-4.5-30-b		3.052	196.8	137.7	30.0	128.7	234.5	291.1	0.292	12.2
T-4.5-30-c		3.013	199.7	139.0	30.4	130.1	257.6	324.0	0.331	15.4

Note: *t*_s_ is the initial thickness of steel, mm; *γ* is the corrosion rate of steel, %; *E*_s_ is the elastic modulus of steel, GPa; *f*_y_ is the yield strength of steel, MPa; *f*_u_ is the ultimate tensile strength of the steel; *μ*_s_ is the Poisson’s ratio of.
